# Fast Fourier Transform IR Characterization of Epoxy GY Systems Crosslinked with Aliphatic and Cycloaliphatic EH Polyamine Adducts

**DOI:** 10.3390/s100100684

**Published:** 2010-01-19

**Authors:** Goran Nikolic, Sasa Zlatkovic, Milorad Cakic, Suzana Cakic, Caslav Lacnjevac, Zoran Rajic

**Affiliations:** 1 Faculty of Technology, University of Nis, Bulevar oslobodjenja 124, Leskovac 16000, Serbia; E-Mails: milorad_cakic@yahoo.com (M.C.); suzana_cakic@yahoo.com (S.C.); 2 Actavis Trading Limited, Djordja Stanojevica 12, Belgrade 11070, Serbia; E-Mail: szlatkovic@info-net.co.rs; 3 Faculty of Agriculture, University of Belgrade, Nemanjina 6, Belgrade, Serbia; E-Mails: ukilaki@eunet.rs or lcaja@agrif.bg.ac.rs (C.L.); zorajic@agrif.bg.ac.rs (Z.R.)

**Keywords:** epoxy resin, FT-IR spectroscopy, polyamine adducts

## Abstract

The use of fast FT-IR spectroscopy as a sensitive method to estimate a change of the crosslinking kinetics of epoxy resin with polyamine adducts is described in this study. A new epoxy formulation based on the use of polyamine adducts as the hardeners was analyzed. Crosslinking reactions of the different stoichiometric mixtures of the unmodified GY250 epoxy resin with the aliphatic EH606 and the cycloaliphatic EH637 polyamine adducts were studied using mid FT-IR spectroscopic techniques. As the crosslinking proceeded, the primary amine groups in polyamine adduct are converted to secondary and the tertiary amines. The decrease in the IR band intensity of epoxy groups at about 915 cm^−1^, as well as at about 3,056 cm^−1^, was observed due to process. Mid IR spectral analysis was used to calculate the content of the epoxy groups as a function of crosslinking time and the crosslinking degree of resin. The amount of all the epoxy species was estimated from IR spectra to changes during the crosslinking kinetics of epichlorhydrin.

## 1. Introduction

Among the different resinous materials utilized in polymeric applications, epoxies exhibit excellent electrical properties, good adhesion to materials having polar groups, low curing temperature, shrinkage on curing, good impact resistance and moisture resistance [1,2]. The development of new epoxy resins has been carried out on two aspects of the epoxy resins. Namely, development and modifications of newer epoxy resins, and their applications in manufacturing composites, coatings, adhesives, paints, varnishes, construction materials and some other advanced fields [3,4].

Epoxy resins crosslinked with aliphatic or cycloaliphatic amines are extensively used in protective coating applications [5]. Since the degree of crosslinking or the chemical conversion is related to the performance and properties of epoxy coatings, several methods have been used to monitor the crosslinking reactions of epoxy/amine resins [5–9]. Among them, fluorescence techniques using molecular probes as extrinsic fluorophores have been applied to monitor the crosslinking process of epoxy/aromatic amines as well as epoxy/aliphatic amines. Non-reactive probes, such as excimer-forming probes [9], viscosity-sensitive probes [10], fluorescence quenching probes [11], polarity probes [12], free volume probes [13], and dual probe-label [14,15], as well as other probes [16–19] have been used to monitor the cure reactions.

Cure reactions of the stoichiometric mixtures of diglycidyl ether of bisphenol A (DGEBA) and two very low molecular weight aliphatic polyether diamines (PED) were studied by using mid and near infrared (IR) spectroscopic techniques [20]. Both Raman [21] and IR micro-spectroscopy [22] have been used to map differences on polymer surfaces. In recent years, attenuated total reflection infrared (ATR-IR) micro-spectroscopy has been applied [23]. High thermally stable epoxy resins, prepared from Schiff base monomer and polymer, were studied by IR, ^1^H-NMR, GPC, DSC, TGA spectral analyses [24]. The molar mass of polyamide block/graft copolymers was determined by size exclusion chromatography (SEC) techniques [25]. New epoxide and cyanate ester resins with an aromatic ester backbone were synthesized and the intermediates were characterized by IR, ^1^H/^13^C-NMR spectroscopic methods [26]. Physical miscibility at ambient and chemical interactions at elevated temperatures between a difunctional epoxy of DGEBA and a linear phenyl-hydroxyl containing polymer, poly (4-vinylphenol), were investigated by DSC, FT-IR, SEM and solid-state ^13^C-NMR [27]. The rates of cure of bisphenol A based epoxy resin and its mixtures with diethylene glycol based epoxy resin with maleic anhydride and BF_3_ etherate accelerator were measured from the dependences of degree of cure with time, using GPC, DSC and IR spectroscopy [28]. A methodology for computer simulation and construction of atomistic models of crosslinked polymer networks has been developed [29]. The methodology has been applied to low molecular weight water soluble epoxy resins crosslinked with different curing agents that are being considered for use as a primer coating on steel.

A search for new improved components for coating systems requires a better understanding of the structure-property relationships of the materials [30]. Although some properties of coating systems can be obtained experimentally, an ability to predict properties of new coatings prior to laboratory synthesis will significantly facilitate new coating design [31,32]. Infrared absorption spectroscopy (IR) provides very valuable information about the chemical structure of the compositions. Besides, IR is available especially for the crosslinking analysis, and it has been used for the estimation of both epoxy and hydroxyl functional groups in polymeric materials. However, measurement of any change of less than several percent of the components is difficult by IR.

**Figure 1.**
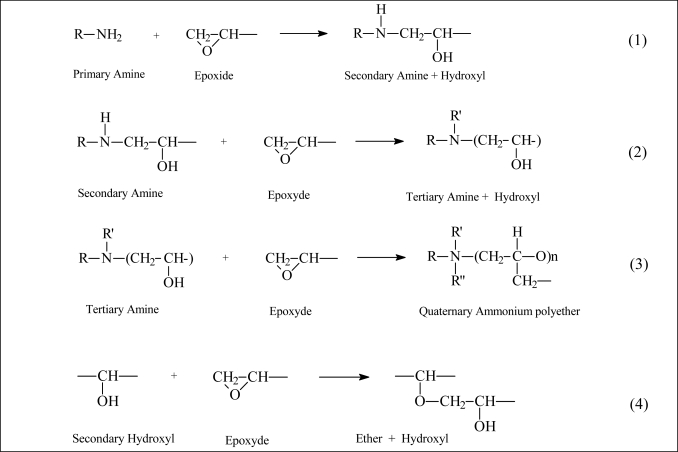
Scheme of epoxy/amine reactions (1–3) and etherification (4) in epoxy/aliphatic amine reactions.

Aliphatic amines are well known quenchers of the aromatic hydrocarbons [33–35]. It has been reported that the quenching of the fluorescence of the aromatic hydrocarbons by aliphatic amines increase with electron-donating ability of the amine groups in the order of R_3_N > R_2_NH > RNH_2_ [33]. Thus, decrease and disappearance in the IR band intensity of NH vibrations as cure proceeds can be expected, since the primary amines become the secondary and the tertiary amine with cure, as shown in Figure 1, in the reactions (Equation 1–3), if the quenching effect is stronger than the intensity enhancing effect of increasing viscosity during cure [36,37]. The fourth reaction (Equation 4, Figure 1), etherification is neglected in epoxy/aliphatic amine reactions [37,38].

The reaction of the primary amine with the epoxide to form a secondary amine (Equation 1) and the further reaction of the secondary amine with the epoxide to form a tertiary amine (Equation 2) are the main chemical reactions that take place (Figure 1). The teriary amine group exerts a catalytic effect and causes the epoxide group to self-polymerize to form a polyether (Equation 3). The hydroxyl groups formed during the ring opening of the oxirane ring accelerate the epoxy-amine reaction, resulting in typical autocatalytic behavior. The basis for this acceleration is a termolecular epoxy/amine/hydroxyl complex. Other possible reactions include homo-polymerization of epoxy resins and etherification between epoxy and hydroxyl groups (Equation 4). However, homo-polymerization of epoxy groups is generally considered negligible in the absence of Lewis acid or base catalysts. For a stoichiometric molar ratio or when amine is present in excess, the etherification reaction is generally insignificant in comparison with the reactions in Figure 1. Moreover, etherification is usually much slower than the amine–epoxy reactions and only becomes significant in epoxy-rich systems when the primary amine is sufficiently depleted [39].

The crosslinking of epoxy resins can be achieved through two different reaction mechanisms, polymerization by addition and by steps [40]. The most common curing process is based on the addition reaction of a hardener. Depending on the crosslinker agent, the final properties of epoxy network are different. When the curing agent is an aliphatic amine, the curing process frequently occurs at room temperature, but it is slow and incomplete. Therefore, the obtained networks present low glass-transition temperatures (Tg) and a high ability to carbonate and to absorb water. On the other hand, epoxy resins cured with aromatic amines generally present good thermal and chemical resistance, while the advantages of anhydride hardeners are their low contraction, viscosity and excellent thermal resistance. The homo-polymerization is another epoxy curing procedure, which consists of polymerization by steps initiated through tertiary amines [41]. Although the reaction mechanism is still a controversy, it is believed that the initiator forms adducts with oxirane groups (initiation). Then this adducts react with themselves and other epoxy rings (propagation). At the end, the initiator regeneration occurs by *N*-alkylation or Hoffman elimination (termination).

In this study, a newer analysis method to measure the distribution of small amounts epoxy functional groups presented in the crosslinked polymers using available equipment was searched. Consequently, the main objective of this research is to monitor the crosslinking reactions by fast IR spectroscopy from the epoxy GY250 resin based diglycidyl ether of bisphenol A, cured with different polyamine adducts (aliphatic EH606 and cycloaliphatic EH637).

## 2. Experimental

### 2.1. Materials

**Figure 2.**
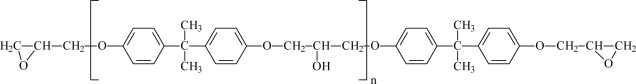
Chemical structure of diglycidyl ether bisphenol A (DGEBA) epoxy resin.

Unmodified medium viscous (η = 9,000–12,000 mPa·s) epoxy resin GY250 based on diglycidyl ether of bisphenol A (DGEBA), supplied by Vantico AG (Switzerland), whose epoxy equivalent weight is 183–189 g/epoxy equivalent, and flash point is 200 °C, was used as received. The aliphatic EH606 and cycloaliphatic EH637 polyamine adducts, supplied by Solutia-Vianova (Austria), whose amine equivalent weight are 100 g/NH equivalent, were used as received. The chemical structure of DGEBA resin is shown in Figure 2.

### 2.2. Characterization of the Polyamine Adducts

Detailed characterization of the polyamine curing agents was done by manufacturer Company: Cytec-Liquid Coating Resins & Additives. Beckopox EH 606 is aliphatic polyamine adduct. It is a relatively low viscous hardener, which can be used with liquid solvent-free epoxy resins. At room temperature such systems have a long pot life of approx. 6–7 hours. Even at higher ambient temperatures, a sufficiently long pot life is exhibited. The hardener can also be used for preparing moldings. In spite of the long pot life, it will not influence the curing speed when preparing moldings, exothermy is very little. Beckopox EH 606 shows the very lowest reactivity of all Beckopox-hardener grades; therefore it is often used as a partner for highly reactive hardeners. Physical properties: H-equivalent weight (f.o.d.) is 100 g/mol. Dynamic viscosity (DIN EN ISO 3219) (100 1/s; 20–29 °C): 2,400–4,400 mPa·s; Amine value (reaction resins) (DIN 16945/5.6) (f.o.d.): 310–350 mg KOH/g; Iodine colour number (DIN 6162) ≤ 3; Density (liquids) (DIN EN ISO 2811-2) approx. (20 °C): 1,03 g/cm^3^; Flash point (Pensky-Martens) (DIN EN ISO 2719) > 100 °C.

Beckopox EH 637 is cycloaliphatic polyamine adduct. It is a modified polyamine with very low viscosity, for use together with liquid epoxy resins for flooring compounds with good chemical resistance and low yellowing tendency. For thin walled laminates a post cure of 2–3 hours at 40–50 °C is necessary prior to deforming. Physical properties: H-equivalent weight (f.o.d.) is 100 g/mol. Dynamic viscosity (DIN EN ISO 3219) (500 1/s; 20–29 °C): 80–120 mPa·s; Amine value (reaction resins) (DIN 16945 / 5.6) (f.o.d.): 300–350 mg KOH/g; Iodine colour number (DIN 6162) ≤ 2; Density (liquids) (DIN 53217-3) approx. (20 °C): 1,00 g/cm^3^; Flash point (Pensky-Martens) (DIN EN 22719) approx. 96 °C.

### 2.3. Epoxy Resin Crosslinking

The first crosslinking process was carried out by addition of the amine hardener EH606 and the amine hardener EH637 in 2 g of the unmodified GY250 epoxy resin (stoichiometric ratio GY250/EH606/EH637 100:25:25). Other studied samples were prepared in different stoichiometric ratio: 100:30:20 and 100:20:30. Stoichiometric mixtures of GY250/EH606/EH637 were mixed using a glass rod and magnetic stirring, followed by crosslinking in a convection oven at 30 °C. At certain time intervals, samples were removed from the oven and IR spectra were taken at room temperature of 25 °C. Samples crosslinked at room temperature were stored in a desiccator.

### 2.4. FT-IR Analysis

A drop of provided mixture was placed between two KBr disks for the mid-IR characterization, using a BOMEM Hartmann&Braun MB-100 instrument, with a 40 scan averaging at a resolution of 2 cm^−1^. For the epoxy grous characterization, the sample was molten between two plain KBr plates separated by a 0.2 mm thick spacer. To assure a fixed path length during crosslinking process, the edge of the samples were covered with high strength epoxy paste. The IR spectra of polyamine adducts films were performed between two quartz plates (5 × 2 × 0.4 cm) using a 0.2 mm thick spacer on the edges, with a Bomem MB-100 spectroscop. The measurement time for each spectrum was 20 s. The epoxy content of resins is frequently expressed as weight per epoxide or epoxide equivalent weight and epoxy functionalities were determined according published methods [4,42].

## 3. Results and Discussion

### 3.1. IR Spectral Analysis of Initial Compounds

The structures of GY250 epoxy resin and used EH polyamine adducts were confirmed by IR spectral analyses. The three investigated components of the polymer system have very different IR spectra. The spectral differences of pure samples can be clearly seen in Table 1. The IR spectra of GY250 reveal the presence of characteristic absorption bands for Ar–C=C–H stretching and bending –CH_2_ and –CH_3_ asymmetrical and symmetrical, –C–Ar–O–C stretching, and epoxy CH_2_–(O–CH–) ring stretching vibration (Table 1). The presence of epoxy groups in IR spectra was proved from the presence of strong bands at 3,056 cm^−1^ (ν_C–H_ epoxy) and 915 cm^−1^ (γ_C–O_ epoxy). The 1,4-substitution of aromatic ring is seen at 830 cm^−1^ for GY250 epoxy resin.

The IR analyses were carried out for both EH606 and EH637 polyamine adducts. The IR spectra reveal the presence of characteristic absorption bands for N–H stretching and bending vibration. The broad doublet peak observed between 3,340–3,200 cm^−1^ is due to the −NH_2_ vibration absorption of amine compounds. Another doublet peak observed between 1,350–1,380 cm^−1^ is due to the presence of isopropyl group vibration absorption. The doublet peak from 1-substitution of aromatic ring is seen at 735 and 698 cm^−1^ for EH637 polyamine adducts. The –C–N group absorption frequency is seen at 1,109 cm^−1^ for EH606 and 1,046 cm^−1^ for EH637. The ether C–O–R is seen at 1,025 cm^−1^. The aromatic –CH, and –C=C– vibrations are seen around 3,030 and 1,605 cm^−1^ region for both the polyamine adducts. The aliphatic –CH_2_ and –CH_3_ vibrations are seen between 3,000 and 2,850 cm^−1^ for both the polyamine adducts (Table 1). The most obvious distinguishing features are that the polyamine adducts spectra contains an intense broad N–H stretching absorption around 3300 cm^−1^, while the resin has an slight sharp band at 3,510 cm^−1^ assigned to the phenyl OH stretching vibration.

### 3.2. IR Spectral Analysis of GY250/EH606/EH637 System

The prepared GY250/EH606/EH637 system was characterized by IR for the sake of confirming its composition (Table 1). The crosslinking of GY250 compound was confirmed by the identification of characteristic absorption peaks. The IR spectrum (not dhown) dispalys a strong broad band in the 3,600–3,200 cm^−1^ region assigned to O–H stretching vibrations. The appearance of the band at 1,638 cm^−1^ indicates the formation of OH groups. A strong bands at 1,605, 1,580, 1,510, 1,455 cm^−1^ are assigned for Ar–C=C–H stretching vibrations. The two bands at 729 and 693 cm^−1^ may be attributed to out of plan bending of aromatic rings. The disappearance of the bands at 3,056 and 915 cm^−1^ indicates the opening of epoxy rings. The appearance of the band at 1,109 cm^−1^ is characteristic for C–N stretching vibrations. The absence of the absorption of epoxy ring and presence of OH group and C–N group confirms the conversion of epoxy group into the corresponding polymer, as well as crosslinking process.

### 3.3. Crosslinking Characterization by IR Spectroscopy

**Figure 3.**
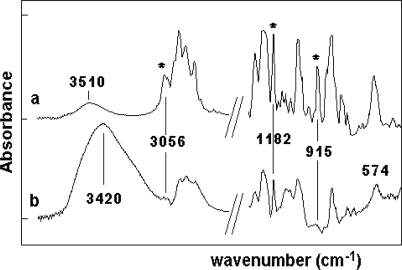
The partial FT-IR spectra of the unmodified GY250 resin (a) and the crosslinked epoxy GY250/EH606/EH637 system after three days (b).

Figure 3 shows the partial mid-IR spectra of GY250/EH606/EH637 system during crosslinking at 30 °C. The peaks of the epoxy group at 3,056 cm^−1^ and 915 cm^−1^ decrease during the crosslinking process, as shown in the IR spectra (Figure 3). Subtraction was carried out to remove the small peak at 3,030 and 910 cm^−1^ in the epoxy peak area [43].

The extent of epoxy reactions of GY250/EH606/EH637 were determined by the peak areas of epoxy peak at 915 cm^−1^ in reference to the peak at 1,182 cm^−1^, which is due to C–O stretching of aromatic ring of DGEBA [20,44].

Epoxy conversion (α_IR_) is thus given by Equation (5):

(5) $$\alpha_{\mathrm{IR}}=1-\frac{\left[\left(A_{915,t}\right)\left(A_{1182,0}\right)\right]}{\left[\left(A_{915,0}\right)\left(A_{1182,t}\right)\right]}$$

where *A*_1182,0_ and *A*_1182,t_ refer to the areas of the reference peak at time zero (0) and after certain time (t), respectively. *A*_915,0_ and *A*_915,t_ are the areas of epoxy peak for uncrosslinked resin and partially crosslinked resin after a certain time, respectively.

**Figure 4.**
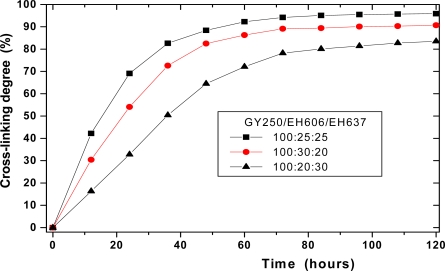
Extent of epoxide reaction in GY250/EH606/EH637 systems followed by mid FT-IR spectroscopy at 30 °C.

The filled symbols (▪) in Figure 4 show the extent of epoxide reaction of GY250/EH606/EH637 as a function of crosslinking time at 100:25:25 stoichiometric ratio. The crosslinking is reasonable, reaching about 94 % crosslinking after 3 days. The filled symbols (♦,▴) in Figure 4 show the extent of epoxy reaction as a function of crosslinking time at 100:30:20 and 100:20:30 stoichiometric ratio, respectively. The crosslinking at 100:30:20 stoichiometric ratio produces about 89 % crosslinking after 3 days, while at 100:20:30 stoichiometric ratio only pushes the reaction to about 78 % crosslinking after 3 days.

The extent of crosslinking reaction of GY250/EH606/EH637 system by the IR peak area of the epoxy group absorbance at 3056 cm^−1^ was verified. The extent of reaction by IR is defined as follows:

(6) $$\alpha_{\mathrm{IR}}=1-\frac{A_{t}}{A_{0}}$$

where *A*_0_ is the peak area at time zero (0), and *A*_t_ is the peak area after certain time (t). By comparison, the exceptionally good agreement of these results with the error being <2% can be observed.

### 3.4. Estimation of the Epoxy Groups Content

The methods described by Zlatkovic *et al.* [4,42,45,46] allowed the calculations of the concentration of the epoxy groups. For each IR spectrum, the ratio of the area of the epoxy group band (attributable to system) to the epoxy group band (attributable to epichlorhydrin) was calculated. Thereby, the content of the epoxy group is determined by IR using the area of the peak at 3,056 cm^−1^ in GY250/EH606/EH637 system during crosslinking process. The content of the epoxy group is determined by linear regression (*r* = 0.9994, *SD* = 0.0142), using the following Equation (7):

(7) P=0.02086+0.00363×Eg

where *P* is the peak area of absorbance at 3,056 cm^−1^ and *Eg* correspond to the epoxy groups content after a certain crosslinking time.

**Figure 5.**
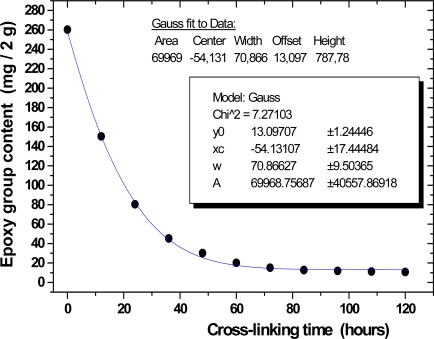
The epoxy group content in GY250/EH606/EH637 system (100:25:25 stoichiometric ratio) during crosslinking process.

Figure 5 shows the concentration of the epoxy groups as a function of crosslinking time for the GY250/EH606/EH637 crosslinking at 30 °C. In the GY250 resin, most of the epoxy groups disappear after three days. There is a minimum concentration of the groups. The concentration of the epoxy groups is 17.1 mg from 2 g of resin. Thus, GY250/EH606/EH637 at 100:25:25 stoichiometric ratio shows faster conversion of epoxy group than those in GY250/EH606/EH637 at 100:30:20 or 100:20:30 stoichiometric ratio.

## 4. Conclusions

A new solvent free epoxy formulation based on the use of aliphatic and cycloaliphatic polyamine adducts as the hardeners was analyzed and characterized by fast FT-IR spectroscopy. IR spectroscopy proved to be a good method for monitoring and assignation of characteristic changes occuring during the crosslinking of epoxy resin. As the crosslinking proceeded, the primary amine groups in EH polyamine adducts are converted to secondary and tertiary amines. The decrease in the IR band intensity of GY250 resin at about 915 cm^−1^ was observed due to more effective quenching of the tertiary amine groups in EH polyamine adducts, in comparison to the IR band at about 1,182 cm^−1^. Moreover, a large decrease in the IR band intensity of GY250 resin at about 3,056 cm^−1^ was observed. Therefore, mid IR spectral analysis was used to calculate the concentration of the epoxy groups as a function of crosslinking time. The amount of all the epoxy species was estimated from mid IR spectra of GY250 in comparison with epichlorohydrin spectra. The GY250/EH606/EH637 system at 100:25:25 stoichiometric ratio betoken relatively good elasticity, adhesive resistance, and low water absorption compared with other aliphatic amine/epoxy resins.

## Figures and Tables

**Table 1.** IR absorption bands of the epoxy GY250 resin, EH polyamine adducts and their crosslinked GY250/EH system.

**IR (frequency, cm^−1^)**	**GY250 Epoxy resin**	**EH606 and EH637 Polyamine adducts**	**GY250/EH System**

Ph-OH str	3,510 weak	-	-
C-OH str	-	-	3,427
-CH-(O-CH_2_) epoxy, str	3,056	-	3,055, disappearance
Ar =C-H str	3,030	3,029	3,026
-NH_2_, -NH str	-	3,340-3,170	-, OH overlay
-NH_2_, -NH bend	-	1,510, 1,495	1,511, disappearance
-CH_2_-, -CH_3_- assym str	2,925, 2,967	2,918, 2,950	2,921, 2,965
-CH_2_-, -CH_3_- sym str	2,855, 2,872	2,850, 2,870	2,852, 2,865
Ar -C-H overtone	2,000-1,600	2,000-1,600	2,000-1,600
-OH bend	-	-	1,638, overlay
Ar -C=C-H str	1,607,1,580, 1,510	1,605, 1,580, 1,510	1,602, 1,581, 1,511
-CH_2_-, -CH_3_- bend	1,455, 1,362	1,458, 1,374	1,460, 1,380
-C-C-O-C- str	1,247, 1,184	1,250, 1,181	1,251, 1,182
-C-N- str	-	1,109, 1,046	1,109
-O-C-C str	1,132	1,081	1,085
-C-O-C- str	1,036	1,025	1,035
CH_2_-O-CH epoxy, bend	915	-	915, disappearance
Ar 1,4 substit. ring and C-O-C (oxirane)	831	829	826
Ar =C-H, C-H,	830, 773	735, 698	729, 693
-C-H, -N-H bend	574, 638	595, 573	550
